# Plasma redox status is impaired in the portacaval shunted rat – the risk of the reduced antioxidant ability

**DOI:** 10.1186/1476-5926-7-1

**Published:** 2008-02-05

**Authors:** Maria-Angeles Aller, Maria-Inmaculada García-Fernández, Fernando Sánchez-Patán, Luis Santín, José Rioja, Raquel Anchuelo, Jaime Arias, Jorge-Luis Arias

**Affiliations:** 1 Surgery I Department, School of Medicine, Complutense University of Madrid, Spain; 2 Human Physiology Department, School of Medicine, University of Malaga, Spain; 3 Psychobiology Laboratory, School of Psychology, University of Oviedo, Asturias, Spain

## Abstract

**Background:**

Portacaval shunting in rats produces a reduction of hepatic oxidant scavenging ability. Since this imbalance in hepatic oxidant/antioxidant homeostasis could coexist with systemic changes of oxidant stress/antioxidant status, plasma oxidants and antioxidant redox status in plasma of portacaval shunted-rats were determined.

**Results:**

Male Wistar male: Control (n = 11) and with portacaval shunt (PCS; n = 11) were used. Plasma levels of the oxidant serum advanced oxidation protein products (AOPP), lipid hydroperoxides (LOOH), the antioxidant total thiol (GSH) and total antioxidant status (TAX) were measured. Albumin, ammonia, Aspartate-aminotransferase (AST), Alanine-aminotransferase (ALT), thiostatin and alpha-1-acid glycoprotein (α_1_-AGP) were also assayed 4 weeks after the operation. AOPPs were significantly higher (50.51 ± 17.87 *vs. *36.25 ± 7.21 μM; p = 0.02) and TAX was significantly lower (0.65 ± 0.03 *vs. *0.73 ± 0.06 mM; p = 0.007) in PCS compared to control rats. Also, there was hypoalbuminemia (2.54 ± 0.08 *vs. *2.89 ± 0.18 g/dl; p = 0.0001) and hyperammonemia (274.00 ± 92.25 *vs. *104.00 ± 48.05 μM; p = 0.0001) and an increase of thiostatin (0.23 ± 0.04 *vs. *0.09 ± 0.01 mg/ml; p = 0.001) in rats with a portacaval shunt. The serum concentration of ammonia is correlated with albumin levels (r = 0.624; p = 0.04) and TAX correlates with liver weight (r = 0.729; p = 0.017) and albumin levels (r = 0.79; p = 0.007)

**Conclusion:**

These findings suggest that in rats with a portacaval shunt a systemic reduction of oxidant scavenging ability, correlated with hyperammonemia, is principally produced. It could be hypothesized, therefore, that the reduced antioxidant defences would mediate a systemic inflammation.

## Background

Portosystemic collateral circulation is a frequent complication of chronic liver disease [1,2]. The portacaval shunted rat is an experimental model of great interest for studying the metabolic alterations related to a portosystemic shunt [3]. Particularly, in this model it has been described that, portal blood flow deprivation (long-term ischemia) may make the atrophic liver more susceptible to oxidant-induced injury because the oxidant scavenging system of the liver decreases [4].

However, recent evidence has shown that the altered redox status in liver disease is not confined to the diseased liver, but that it is a systemic phenomenon involving extrahepatic tissues [5]. So, the determination of oxidant and antioxidant plasma levels in portacaval shunted rats could broaden the knowledge of the systemic pathophysiological mechanisms, which are activated by the systemic bypass of the portal blood flow.

This study has been carried out to determine serum advanced oxidation protein products (AOPP), lipid hydroperoxides (LOOH), total serum antioxidants (TAX), total thiols and albumin as markers of the plasma redox status.

## Results

### Body and liver weights

Rats with portacaval shunt (PCS) show a body weight (BW) decrease (p < 0.001) during the 4 weeks of postoperative evolution. Liver weight (LW) and LW/FBW ratio are also inferior (p < 0.001) in rats with PCS in relationship to control rats (Table 1).

**Table 1:** Body parameters. Initial body weight (IBW), final body weight (FBW), body weight increase (BWI), liver weight (LW) and liver weight/body weight ratio (LW/FBW) in control rats and in rats with portacaval shunt (PCS) at 4 weeks of evolution.

**Group**	**IBW (g)**	**FBW (g)**	**BWI (g)**	**LW (g)**	**LW/FBW × 100**
**Control (n = 11)**	221.55 ± 5.01	267.82 ± 5,69	46.82 ± 6.35	7.48 ± 0,40	2.79 ± 0.14
**PCS (n = 11)**	237.64 ± 12.96	212.00 ± 22.19***	-25.09 ± 25.05***	4.03 ± 0.63***	1.90 ± 0.24***

Data presented as: Mean ± SD. ***Statistically different from the control group (p < 0.001).

### Hepatic liver function assays

Aspartate-aminotransferase (AST) (p = 0.004), alanine-aminotransferase (ALT) (p = 0.0001), ammonia (p = 0.0001) and thiostatin (p = 0.0001) serum levels are higher in PCS-rats compared to control rats. On the contrary, albumin (p = 0.0001) and α_1_-acid glycoprotein (α_1_-AGP) (p = 0.04) are lower in PCS-rats (Table 2).

**Table 2:** Biochemical data. Aspartate-aminotransferase (AST), alanine-aminotransferase (ALT), AST/ALT ratio, ammonia, albumin, thiostatin and α _1 _acid glycoprotein (α _1_-AGP) serum concentrations in control rats and in rats with portacaval shunt (PCS), 4 weeks after the operation.

**Group**	**Control (n = 11)**	**PCS (n = 11)**	**p-value**
AST (IU/L)	65.72 ± 11.19	134.50 ± 67.60	p = 0.004
ALT (IU/L)	29.36 ± 5.90	65.33 ± 27.27	p = 0.0001
AST/ALT	2.35 ± 0.08	2.38 ± 1.58	NS
Ammonia (μmol/L)	104.00 ± 48.05	274.00 ± 92.25	p = 0.0001
Albumin (g/dl)	2.89 ± 0.18	2.54 ± 0.08	p = 0.0001
Thiostatin (mg/ml)	0.09 ± 0.01	0.23 ± 0.04	p = 0.001

Data presented as: Mean ± SD. NS = non-significant difference.

### Redox status

**Figure 1:**
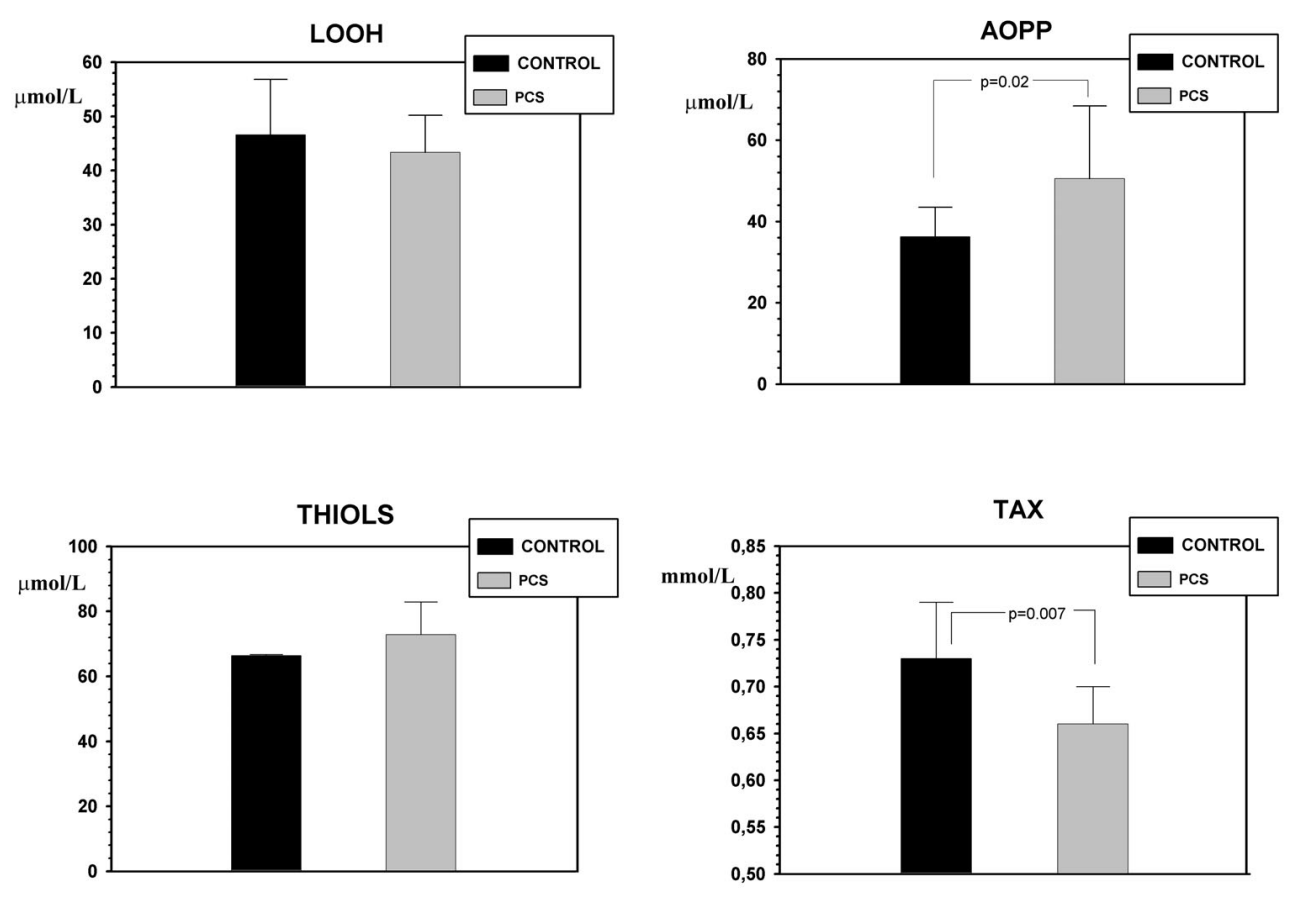
**Redox status in control rats and in rats with portacaval shunt at 4 weeks of evolution**. Duplicate (TAX, AOPP and THIOLS) and triplicate (LOOH) assays in control (n = 11) and portocaval shunt (PCS) (n = 11) rats, except for TAX in which one PCS value was excluded. The results are expressed as mean ± SD. AOPP: serum advanced oxidation protein product; LOOH: serum lipid hydroperoxides; TAX: serum total antioxidant; THIOLS: total plasma thiols.

The serum advanced oxidation protein product (AOPP) level increases (p = 0.02) whereas total antioxidant status (TAX) decreases (p = 0.007) in portacaval shunted rats in relation to control rats. The serum concentrations of lipid hydroperoxides (LOOH) and total thiols do not change in PCS-rats (Figure 1).

### Correlation between liver function parameters and serum redox status

**Figure 2:**
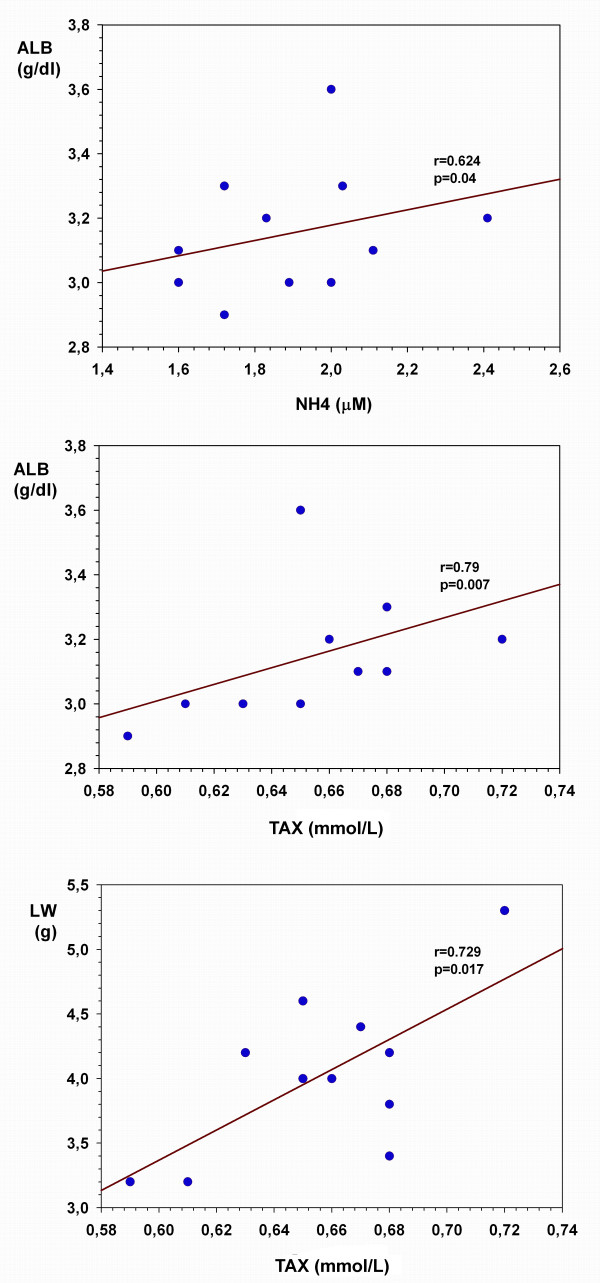
**Ammonia and total antioxidant status**. Ammonia and serum total antioxidant (TAX) status are correlated with albumin serum levels. TAX also correlated with hepatic atrophy in portacaval shunted-rats 4 weeks after the operation.

The serum concentration of ammonia correlates with albumin levels (r = 0.624; p = 0.04) and TAX correlates with liver weight (r = 0.729; p = 0.017) and albumin levels (r = 0.79; p = 0.007) (Figure 2).

## Discussion

The results reported in this study show a significant decrease of the TAX, associated with an increased AOPP plasmatic level of portacaval-shunted rats. The considerable decrease in TAX levels in long-term (4 weeks) portacaval shunted-rats suggest that a weakening of the antioxidative barrier of the body exists, perhaps as a consequence of the increased systemic oxidative stress produced by the portosystemic shunting in this experimental model.

Oxidative stress, in general, is the overpowering of the antioxidative defence system by the oxidative system [6]. A number of diseases, including liver disease [5], are associated with an imbalance between oxidant stress and antioxidative defence mechanisms that favour the former [5,7]. Oxidative stress is produced by free radicals, i.e., reactive oxygen species (ROS) and reactive nitroxy species (RNOS) and if they are not removed or neutralized, react with lipids, proteins, and nucleic acids, damaging the cellular functions and eventually causing cell death [5,6,8]. Both the excessive oxidative stress and the reduced antioxidant ability could participate in the imbalance between the oxidant stress and antioxidative defence mechanism, which is produced in the rats with portacaval shunt.

Chronic liver ischemia derived from the portal blood flow bypass in the rat impairs oxidant scavenging, but does not impair the oxidant generating systems of the liver [4]. However, the sources of ROS and RNOS in liver diseases can be subdivided into intrahepatic and extrahepatic. Particularly, the extrahepatic oxidative stress is considered a systemic phenomenon involving extrahepatic tissues [5] and mainly portal circulation [5,9]. In rats with portal vein stenosis and portosystemic collateral circulation, the existence of a causal relationship between oxidative stress and the hyperdynamic circulation developed has been accepted [9]. Since the hepatocellular injury is not a feature of this animal model it has been proposed that oxidative stress originates from the portal circulation and not the diseased liver [5,9]. Furthermore, portacaval shunted rats also develop a hyperdynamic splanchnic circulation related to portosystemic shunting [10-12]. Therefore, in this experimental model the hyperdynamic splanchnic circulation or mesenteric hyperemia could also be associated with intestinal oxidative stress. It has been proposed that the chronic hypoxemia of the intestinal mucosa related to vascular congestion could be an etiologic key factor in the production of bacterial translocation because the enterocytes would suffer injury by oxidative stress [13,14]. Moreover, NO-overproduction could represent an adaptive mechanism of the endothelium in response to chronic increases in flow-induced shear stress [15-18]. NO reacting with ROS, such as O_2_^-^., can also induce the peroxynitrite ion (ONOO^-^) hyperproduction [19]. Intestinal oxidative stress could participate through this mechanism in the production of increased plasmatic levels of AOPP in rats with a portosystemic shunt.

Protein oxidation products have increasingly been used as markers instead of lipid peroxidation products in demonstrating oxidative stress [20]. A novel oxidative stress marker of protein, referred to as AOPP was developed in plasma [21]. Furthermore, AOPP oxidation of plasma thiol groups, termed "thiol stress," is quantitatively the major manifestation of protein oxidation [22]. Since AOPP is not only a marker of oxidative stress, but also acts as an inflammatory mediator [23-27] the knowledge of AOPP pathophysiology in this experimental model could provide valuable information with respect to the relationship between oxidative stress and the inflammatory response related to a portosystemic shunt. In this regard, since the liver and the spleen play important roles in the elimination of AOPP [28], the apoptosis and liver atrophy after portacaval shunting in the rat [29-31] could induce its decreased plasma clearance, thus favouring its increased plasmatic levels.

The marked plasmatic levels increase of thiostatin and the hypoalbuminemia in rats with a portosystemic shunt may be involved in the acute phase changes associated with a systemic inflammatory response [32,33]. The proteins acting as acute phase proteins differ from humans to animals and from one species to another. In the rat, thiostatin and α_1_-acid glycoprotein (α_1_-AGP) are among the major positive acute phase proteins while albumin reacts as a negative acute phase protein [34]. Thiostatin is a plasma proteinase inhibitor protecting against proteolytic auto-degradation [33]. Therefore, the synthesis of thiostatin benefits from the metabolic priority during decreased functional liver mass caused by the portosystemic shunt. However, α_1_-AGP does not increase in these animals. Since it is considered that α_1_-AGP prevents gram-negative infections [34] and has anti-inflammatory functions [35], rats with portacaval anastomoses would lose an essential component in nonspecific resistance to infection and inflammation.

Albumin plasma levels correlate with the TAX and with the hyperammonemia in portosystemic shunted rats. Albumin is a powerful extracellular antioxidant [36] and its decreased liver synthesis after portacaval shunt reduces its antioxidant functions. However, albumin synthesis increases when ammonia levels are higher. This could represent an attempt of compensating the deleterious metabolic effects caused by ammonium.

In rats with a portosystemic shunt, the acute-phase response could be associated with oxidative stress, as well as with inflammation. Particularly, IL-6, the major stimulator of most acute phase proteins, is primarily produced by Kupffer cells [37,38]. Upregulation of this cytokine may be related to the enhanced respiratory burst activity of Kupffer cells leading to the redox activation of NF-κB [39-41]. This compensatory response has already been described in order to re-establish homeostasis in the liver and extrahepatic tissues exhibiting oxidative stress [38].

Another metabolic feature that has been shown to be upregulated, though not always, due to a lack of oxygen or oxidative stress, is the antioxidant system [42]. It has been shown that the portosystemic bypass in the rat reduces the oxidant scavenging system of the liver with a significant reduction of superoxide dismutase and xanthine-dehydrogenase [4]. Furthermore, in the present study, the TAX (i.e., the fraction of antioxidant pool available for further anti-ROS activity) is significantly lower in portacaval shunted rats compared to control rats. These results may mean that a portosystemic shunt, including hyperdynamic circulatory syndrome and acute-phase response, has its own effect on lowering TAX. Since oxidative stress exhausts the antioxidative pool of the body, TAX could also decrease [8,42]. However, the ROS overproduction after portacaval shunting is not excessive, and indeed a plasmatic increase of lipid peroxidation is not produced, therefore it can be suspected that the novo antioxidant synthesis is reduced. If so, the reduction of the systemic antioxidant activity makes the organism susceptible to oxidant-induce multi-organ injury because a normal ROS production could be indeed a potential cause of oxidative stress when an antioxidative deficit coexists [6,42-44].

Since the existence of an anti-inflammatory redox-oxidant revolving axis has been suggested [43], in rats with portosystemic shunt, it could also be considered that the reduction of antioxidant ability would represent the mediator signal for the evolution and perpetuation of the inflammatory process that is often associated with the condition of oxidative stress, which involves gene regulation [43,45]. Thus, the altered redox homeostasis in this experimental model would be one of the hallmarks of the processes that regulate gene transcription in oxidative-stress-mediated inflammation [8,43,45]. If so, we could call it: "reduced antioxidative defence-mediated inflammation."

The decrease of the antioxidant protection in rats with portacaval shunt, evidenced by lower TAX and hypoalbuminemia, is noteworthy since it is correlated with hyperammonemia. This correlation suggests that, in this experimental model, the grade of insufficient antioxidant-mediated inflammation would be involved in a particular metabolic alteration related to the portosystemic shunt, as is the ammonia hyperproduction. Hyperammonemia is considered a key etiopathogenic factor in the development of hepatic encephalopathy [46-49]. Although, ammonia is believed to be responsible for the neurological abnormalities associated with hepatic encephalopathy, growing evidence supports the view that glutamine, synthesized from glutamic acid and ammonia, plays a major role in the deleterious effects of ammonia [49] and induce oxidative stress [46-48]. In turn, L-glutamic acid is also a precursor of the antioxidant glutathione [5,42,50,51]. Thus, hyperammonemia could be added as an etiopathogenic factor of the oxidative stress-mediated inflammation pathway that induces the portosystemic shunt [48,49].

## Conclusion

The decreased liver antioxidant activity in portacaval shunted rats could potentiate the oxidative stress. In turn, the increased synthesis of acute phase proteins by the liver, since their anti-enzymatic ability, would attempt to balance the enzymatic stress in this experimental model.

## Methods

### Animals

Male Wistar rats, with weights ranging from 230 to 270 g, from the *Vivarium *of the Complutense University of Madrid, were used. The animals were fed a standard laboratory rodent diet (rat/mouse A04 maintenance diet, Panlab, Spain) and water *ad libitum*. They were housed in a light/dark-controlled room, with an average temperature (22 ± 2°C) and humidity (65–70%) in groups of three to four animals.

The experimental procedures and facilities complied with the requirements of Commission Directive 86/609/EEC (The Council Directive of the European Community) concerning the protection of animals used for experimental and other scientific purposes. The National legislation, in agreement with this Directive, is defined in Royal Decree n° 1202/2005.

### Surgical technique of portacaval shunt

The animals were anesthetized by i.m. injection of ketamine (100 mg/Kg) and xylacine (12 mg/Kg). The end-to-side portacaval anastomoses (PCA) was performed according to a modified [29] Lee's technique [52,53]. In brief, the intestinal loops are retracted to the animal's left and covered with saline wet gauze to expose the inferior vena cava (IVC) and the portal vein (PV). The dissection and vascular anastomoses were done by a microsurgical technique with the aid of an operative microscopy (Zeiss, OPMI-1; 12 × 5). The IVC was dissected between the hepatic parenchyma and the right renal vein. The PV was individualized from the proper hepatic artery and the gastroduodenal vein was dissected and sectioned between ligatures (silk 7/0). The infrahepatic IVC was clamped with two microclips and an elliptical venotomy (3 × 2 mm) was performed on its anterior wall. The PV was then ligated and sectioned in the liver hilum and clamped in its confluence with the splenic vein. Nylon (9-10/0) was used to perform the end-to-side portacaval anastomoses. The midline abdominal incision was closed in two layers using a continuous running technique with an absorbable suture (polyglycolic acid) and 3-0 silk. Analgesia was maintained with buprenorphine (0.05 mg/kg/8 h s.c.) during the first 48 hours after the operation.

The animals were sacrificed by exsanguination 4 weeks after the operation. Hepatic tissue was excised and rapidly frozen in liquid nitrogen. Frozen livers were stored in labelled containers at -80°C for posterior molecular studies and metabolic determinations.

### Biochemical blood assays

Serum levels of albumin, total proteins, AST and ALT were determined by routine laboratory methods using a COBAS MIRA autoanalyzer according to the manufacturer's instructions (HORIBA ABX diagnostic, Montpellier, France). Plasma ammonia was immediately measured by glutamate dehydrogenase enzyme assay on a clinical analyzer (COBAS MIRA autoanalyzer; Products: BIOLABO SA, Maizy, France). Rat alpha-1-Acid Glycoprotein (alpha-1-AGP) and thiostatin serum levels were assayed by ELISA (Life Diagnostics, Inc, USA)

### Total antioxidant status

The total antioxidant capacity of serum was estimated in duplicate using the commercial kit 'Total Antioxidant Status' (Randox, UK), adapted to the Cobas Mira autoanalyser, which measures at 600 nm the formation of the radical ABTS^+ ^using the Reagent ABTS^® ^in the presence of H_2_O_2 _and peroxidase [54]. The method was calibrated using the TROLOX standard included in the kit.

### Determination of plasma sulfhydryl groups

Plasma sulfhydryl (-SH) groups were measured in duplicate by using Ellman's reagent, 5,5'-dithiobis-(2-nitrobenzoate) (DTNB), adapted to Cobas Mira [55]. Ten μl of plasma were mixed with 200 μL of 0.1 M Tris buffer, containing 10 mM EDTA, pH 8.2. The absorbance at 405 nm, given by the plasma alone, was subtracted from that obtained from the same sample 10 minutes after adding 8 μL of 10 mM DTNB. A blank containing only DTNB was also included, and -SH concentration was calculated by using a standard curve of glutathione. Thiol levels were expressed in μmol/L plasma. Intra- and inter-assay variation coefficients were 1.2% and 6%, respectively.

### Evaluation of plasma AOPP

Plasma AOPP were evaluated in duplicate by using a microassay adapted to Cobas Mira according to Matteucci et al [56] and based on the original method of Witko-Sarsat et al. [21]. Briefly, 10 μl of plasma or chloramine-T (ch-T) standard solutions (400 – 6.25 μmol/l) were placed in each well of the Cobas Mira autoanalyser. Then 200 μl of the reaction mixture was added, consisting of 81% phosphate buffer solution (PBS), 15% acetic acid and 4% 1.16 mM potassium iodide. The absorbance was read at 340 nm (the blank contained PBS instead of plasma). AOPP concentration was expressed as ch-T equivalents. Intra- and inter-assay variation coefficients were 1% and 5%, respectively.

### Evaluation of plasma lipid hydroperoxides

Lipid hydroperoxides (LOOH) were evaluated in triplicate by the FOX2 reagent (Ferrous Oxidation) automated by Arab & Steghens [57] and adapted to Cobas Mira (wavelength 600 nm) for studying lipid peroxidation in serum samples. Xylenol orange (180 μl – 167 μM), the first reagent, was added after to the sample (25 μl). The first optical reading was recorded before adding 45 μl of 833 μM iron II D-gluconate. LOOH was calculated using a standard curve of tert-butylhydroperoxide and LOOH levels were expressed in μmol/L serum. Intra- and inter-assay variation coefficients were 3% and 8%, respectively.

### Statistical analysis

Statistical analyses were performed using SPSS software (Statistical Package for the Social Sciences, version 14.00). The results are expressed as mean ± standard deviation (SD). Student's t test for independent data was used to compare the different variables between the two groups of animals. The relationship between the biochemical serum parameters were verified using the Pearson coefficient correlation. A p-value of less than 0.05 was considered significant.

## Abbreviations

AST: aspartate-aminotransferase ; ALT: alanine-aminotransferase; α_1_-AGP: alpha-1 acid glycoprotein; AOPP: advanced oxidation protein products; BW: body weight; ch-: chloramine-T; DTNB: 5,5'-dithiobis-(2-nitrobenzoate); FBW: final body weight; FOX2: ferrous oxidation; GSH: reduced glutathione; IL: interleukin ; IVC: inferior vena cava ; LOOH: lipid hydroperoxides; LW: liver weight; LW/BW: liver weight to body weight ratio; NF-κB: nuclear factor kappa beta; NO: nitric oxide; ONOO^-^: peroxynitrite ion; PBS: phosphate buffer solution; PCA: portacaval anastomoses; PCS: portacaval shunt; PV: portal vein; ROS: reactive oxygen species; RNOS: reactive nitroxy species; TAX: total antioxidant status/capacity of the serum; TNF-α: tumor necrosis factor alpha.

## Competing interests

The author(s) declare that they have no competing interests.

## Authors' contributions

MIGF, FSP, LS, JR, RA, MAA and JLA performed most of the experiments and provided assistance for the preparation of the manuscript. MAA, MIGF, JLA and JA participated in the design of the study and prepared the manuscript. All authors have read and approved the content of the manuscript.
